# An assessment of the replicability of a standard and modified sanitary risk protocol for groundwater sources in Greater Accra

**DOI:** 10.1007/s10661-018-7174-5

**Published:** 2019-01-10

**Authors:** W. Yentumi, M. Dzodzomenyo, K. Sashie-Doe, J. Wright

**Affiliations:** 1 School of Public Health, University of Ghana, PO Box LG13, Legon, Accra, Ghana; 2 Geography and Environment, University of Southampton, Highfield, Southampton, SO17 1BJ UK

**Keywords:** Sanitary risk inspection, Inter-observer agreement, Groundwater, Replicability, Ghana

## Abstract

Sanitary risk inspection, the systematic observation of contamination hazards, is often used to manage safety of water sources such as wells and boreholes. However, the replicability of sanitary risk inspections undertaken by different observers has not been studied. This study aimed to assess the replicability of sanitary risk inspections by two different observers in urban and peri-urban neighbourhoods of Greater Accra, Ghana. Two observers independently used a standard protocol to record contamination hazards around 62 groundwater sources, additionally recording urban-specific hazards such as damaged sewage pipes via a modified protocol. We calculated risk scores as the proportion of hazards observed at each source, separately for each observer. Linn’s concordance correlation coefficient indicated very high agreement between the two observers’ risk scores (*n* = 62; *c* = 0.949, 95% confidence limits 0.917–0.968). However, risk scores from urban-specific observations were uncorrelated with those from the standard protocol (*r* = 0.11, *p* = 0.41 for observer 1; *r* = 0.16, *p* = 0.22 for observer 2). Ours is the first study of replicability of sanitary risk observations and suggests high inter-observer agreement. However, urban contamination hazards were not captured using the standard protocol. In the future, assessment of inter-observer agreement and observations of urban-specific hazards could be incorporated into nationwide or regional sanitary risk surveys.

## Introduction

A sanitary risk inspection is a structured observation checklist used to identify potential sources and pathways for faecal contamination in and around water sources (World Health Organization 1997). Sanitary risk inspection is typically used with community water sources such as hand-dug wells, springs, rainwater systems, and boreholes, rather than a piped supply system. Individual observation items typically include hazards relating to the water supply system itself, such as a missing cover on a well, but also hazards in the immediately surrounding area, such as the proximity of pit latrines. Typically, the number of individual hazards observed at a given source is summed to provide a measure of the overall risk of contamination at that source. The observed hazards can also be used to plan appropriate source remediation, such as repairs to concrete aprons or fencing surrounding wells.

Despite the use of sanitary risk inspection protocols for almost 30 years, several issues affect their use. Firstly, it is unclear whether sanitary risk observations are replicable and whether two observers visiting the same source would consistently observe the same hazards. The robustness of quantitative metrics such as sanitary risk scores is often assessed via two concepts, namely validity (the extent to which the metric measures the intended phenomenon) and reliability (the consistency with which measurements are made) (Heale and Twycross 2015). Key attributes of reliability include stability, the extent to which repeated measurements using the same instrument produce consistent results, and equivalence, the extent to which different users generate consistent results when following the same measurement protocol (Heale and Twycross 2015). Although these concepts are well established in public health and social sciences, they are seldom applied in developing countries (Bolarinwa 2015). To our knowledge, the concept of reliability has not been previously applied to sanitary risk inspection.

Secondly, sanitary risk inspection originated in rural communities, where the low housing density generally means it is relatively straightforward to observe hazards surrounding a given source. However, particularly in sub-Saharan Africa, the use of groundwater sources is comparatively common (Lapworth et al. 2017) in urban and peri-urban areas. In these areas, not only does the density of housing mean that it may be difficult to observe surrounding hazards such as onsite sanitation, but there may also be additional hazards in urban areas such as broken storm drains, leaky sewerage pipes, and industrial facilities that do not exist in rural areas (Okotto-Okotto et al. 2015). Since urban sanitary risk protocols have not always been adapted to include such hazards specific to urban environments (Mushi et al. 2012), evidence on their prevalence remains limited.

Thirdly, because of variation in hydrogeology, the soil matrix, and surface topography, it has proved difficult to identify minimum safe distances between hazards such as water sources. Finally, the validation of such observation protocols is problematic. Whilst many studies have sought to compare the presence of individual hazards and/or overall sanitary risk scores to faecal indicator bacteria counts, results have often been mixed. Perhaps because of the transient nature of contamination in some water supply systems, some studies have found little or no relationship between observed sanitary risk and faecal indicator bacteria (Luby et al. 2008; Ercumen et al. 2017; Parker et al. 2010; Snoad et al. 2017), particularly when looking at individual source types (Misati et al. 2017), whilst others have found stronger relationships (Wright et al. 2013; Howard et al. 2003; Dey et al. 2017). Low reliability in sanitary scores is a possible explanation for the weak relationship that has sometimes been observed between sanitary risk scores and faecal contamination of source water, but as noted above, there are no studies to date examining the reliability of sanitary risk protocols.

This study seeks to address two of these issues in some case study communities in urban and peri-urban Greater Accra. We firstly seek to assess the replicability of observations of individual hazards, through repeat water source visits by different observers on the same day. Secondly, we aim to assess the extent to which additional observation items, specifically introduced to reflect hazards in urban and peri-urban environments, explain water source contamination as measured by faecal indicator bacteria. As a subsidiary objective, we also examine the relationship between sanitary risk and time elapsed since water point installation, examining the impact of inter-observer disagreement on this relationship.

## Methods

### Sample selection and study areas

The sample size was determined based on a least squares regression of overall sanitary risk score (i.e. proportion of hazards on the inspection checklist observed at a given site) versus *Escherichia coli* counts in samples taken from water points. Based on a similar study of groundwater sources in urban Kenya (Wright et al. 2013), an effect size (Cohen’s *f*^2^) of 0.15 for such a model was assumed. With power of 0.8 and alpha = 0.05, we estimated that a minimum sample size of 54 water points was required to detect such an effect via a regression model with a single covariate using an online tool (Soper 2017) implementing Cohen’s approach to sample size estimation (Cohen 1988).

**Fig. 1:**
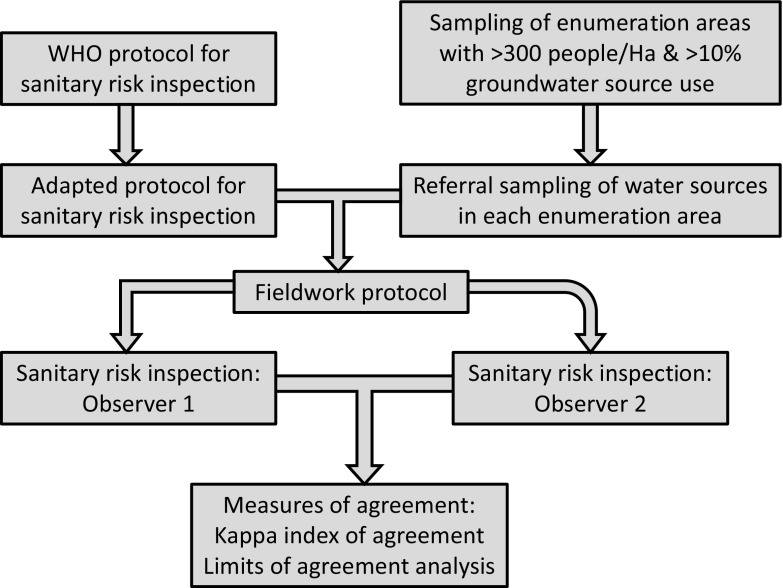
Overview of the study design to assess replicability of sanitary risk inspection observations

**Fig. 2:**
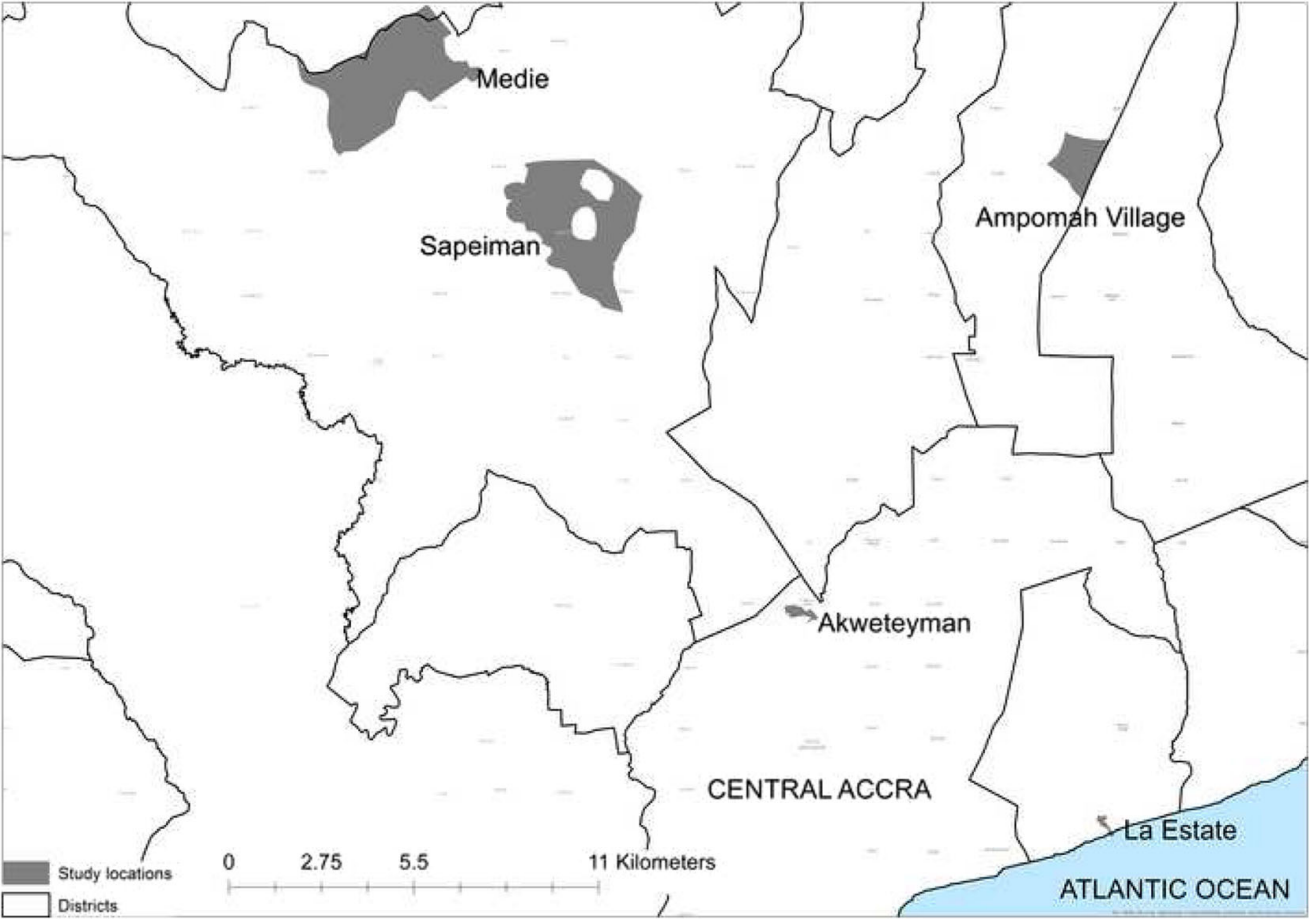
Locations of study site urban and peri-urban neighbourhoods in the Greater Accra region, Ghana (source of district boundaries: the GADM project, https://gadm.org/index.html)

Following determination of sample size, an overview of the study design is provided in Fig. 1. Urban or peri-urban areas reliant on groundwater were selected by identifying enumeration areas in the Greater Accra region with high population densities (> 300 people/Ha) and with greater than 10% of households using point groundwater sources (springs, wells or boreholes) as the main drinking water source using 2010 population census statistics. Five enumeration areas were then selected via simple random sampling from this list. The selected enumeration areas are shown in Fig. 2. These were Sapeiman, a peri-urban area in Ga West District; Medie, a peri-urban area in Ga West District; Akweteyman in the Achimota area of urban Accra Metropolitan Area (AMA), La in the La Estates area also in the AMA and Ampomah village, a peri-urban area in the Adentan municipality in La Nkwantanang-Madina District. According to a 1:100,000 hydrogeological map of the area, the Sapeiman and Medie sites lay on migmatitic granite, whilst the geology of the remaining sites comprised paragneiss sediments with minor schists.

In Ga West district, the 2010 population census (Ghana Statistical Service 2014a) indicated 63.2% of households used sachet water for drinking and only 7.1% boreholes. However, 25.8 and 11.7% respectively used water from boreholes and protected wells for other domestic uses. Sanitation facilities were typically water closets (29.7%), pit latrines (28.9%) or ventilated improved pit latrines (22.6%). Most (56.5%) had their solid waste collected, though some (20.9%) burned their waste. Household characteristics in the remaining study districts were broadly similar, with for example 12.3 and 8.7% respectively using protected well and borehole water for other domestic purposes in La Nkwantanang-Madina District (Ghana Statistical Service 2014b).

Within each enumeration area, a chain referral process was then used to locate groundwater point sources. Once a water point had been identified, the informant was asked to identify one or more neighbouring water points, with the process continuing until no new water points were being identified. All groundwater point sources (both boreholes and wells) identified in this way were included in the study.

### Development of observation protocol

Sanitary risk observation items for wells and boreholes were drawn from the exemplar protocols provided by the WHO (World Health Organization 1997). We then added observable contamination hazards potentially found in urban and peri-urban areas including:

- Presence of ‘flying toilets’, or defecation in polyethylene bags (Tumwebaze et al. 2013), an excreta containment option for urban households lacking sanitation or diapers, also a growing waste management issue in low and middle income countries (Reese et al. 2015)
- Locations of storm drains and blockages to storm drains, both associated with contamination of shallow urban groundwaters (Wakida and Lerner 2005; Kulabako et al. 2007)
- Locations of sewerage pipes and leaky sewers (Wakida and Lerner 2005), associated with shallow urban groundwater contamination in the USA (Lee et al. 2015)

This resulted in two sets of observable hazards at and surrounding water points—those derived from the WHO protocol, and a supplemental set of hazards specific to urban or peri-urban areas.

### Sanitary risk observations

Sanitary risk observations were made by two authors (KSD, WM), both of whom were studying for educational qualifications at the time of the study. Both observers had a public health background, but neither had water and sanitation-related expertise or experience prior to the study. Both were actively involved in the development of the protocol, meeting frequently to review and agree on the interpretation of the protocol prior to the field survey. To ensure the personal safety of the field team, both observers visited each water source together to obtain informed consent to participate in the study from the well owner or manager. Informed consent was obtained from all individual participants included in the study. Whilst one (WM) interviewed the well owner, the second observer (KSD) made observations of hazards around water points based on the protocol. Thereafter, the first observer then recorded observable hazards, whilst the second observer recorded depth to water table and took a water sample for subsequent microbiological testing. Where possible, both observers separately used a 100-m tape to record distances to hazards, otherwise estimating these by eye. One observer (WK) interviewed the water point owner/manager prior to making observations, asking about each source’s history, management and groundwater use and subsequent treatment; the second did not interview the water point owner/manager. Fieldwork took place between 14 March 2017 and 29 March 2017.

### Analysis

Prior to analysis, we grouped the observation items into three classes: transient hazards that might appear or disappear during the course of a day; hazards that might be difficult to observe, either because of settlement density or because they might require inspection of a well shaft for example and hazards that fell in neither of the other two categories. We then cross-tabulated the two sets of observations on each contamination hazard, calculating the kappa index of agreement (McHugh 2012) for each one, so as to correct for chance agreement. We then examined the agreement indices for each of the three groups of observation items, calculating the mean value of the kappa index of agreement for observations in each group.

Sanitary risk scores were calculated based on both sets of sanitary risk observations, separately for the standard observation items, for the observation items specific to urban/peri-urban environments and for the two sets of items combined. Scores were calculated as the proportion of hazards present out of those observable. Where an item was either not applicable (for example where a source lacked a hand pump) or not observable, it was thus excluded from the risk score calculation. We then computed mean risk scores across the two observers and the difference in risk scores, examining concordance between risk scores. We calculated the concordance correlation coefficient and Bradley-Blackwood *F* test for equality of means and variances (Bradley and Blackwood 1989) using the *concord* utility in Stata version 13.1 (StataCorp 2013). Given inequality in means and variances, we used the Stata *batplot* command to produce Bland and Altman plots, thereby accounting for the relationship between levels of bias and/or precision and mean risk score across the two observers. To assess whether hazards associated with the urban environment were implicitly captured via the standard WHO observation protocol, we calculated Pearson’s correlation coefficient between the standard WHO-based sanitary risk scores and the scores derived from urban/peri-urban-specific observation items only. To examine progressive improvement in observation replicability with experience and possible observer fatique, we examined the relationship between the sequence in which water points were visited (first to last) and the difference between the two observers’ sanitary risk scores, both graphically and using Spearman’s rank correlation. Finally, to examine the impact of inter-rater disagreement on inferential analysis concerning the prevalence of contamination hazards, we examined the relationship between water point age and sanitary risk score using both one-way Anova and boxplots.

Ethical approval for the study was granted by the ethics committees of the Noguchi Memorial Institute for Medical Research, University of Ghana (ref: 106/15-16) and the Faculty of Social, Human, and Mathematical Sciences, University of Southampton, UK (ref: 21417).

## Results

### Prevalence of observable hazards

All water point owners/managers selected for the study agreed to participate. This resulted in sampling of 3 boreholes and 59 wells. Fifty-six of these water points were constructed by private contractors, three by non-governmental organisations, with the installer of the remaining water points unknown. Most water points (40; 65.6%) were constructed between 1 and 10 years previously.

**Fig. 3:**
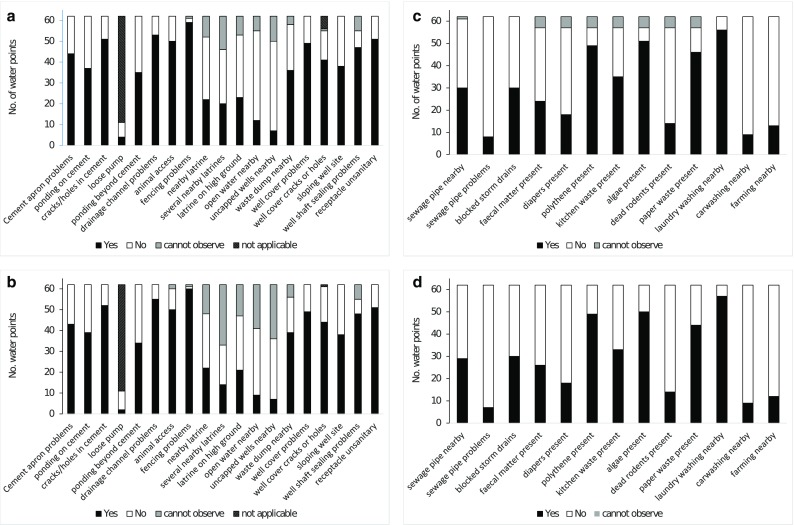
Proportion of contamination hazards observed at 62 groundwater sources in the Greater Accra Region, based on **a** standard WHO-derived observation items, observer 1; **b** standard WHO-derived items, observer 2; **c** observation items specific to urban/peri-urban environments, observer 1; and **d** observation items specific to urban/peri-urban environments, observer 2

Figure 3 shows the prevalence of contamination hazards at the water points, using both the standard observation protocol and that adapted for urban and peri-urban areas. Common problems identified via the standard protocol included inadequate sealing of well shafts, lack of adequate fencing to prevent animals from accessing sources, unsanitary receptacles for extracting groundwater and missing or faulty drainage channels for excess water. The most widespread urban hazards were uncollected solid waste (e.g. plastics, paper), but other hazards such as blocked storm drains and diapers close to water points were relatively common.

### Replicability of hazard scores

Table 1 shows the level of agreement between the two observers for the different hazards. There was moderate agreement (kappa index of agreement between 0.6 and 0.79 (McHugh 2012)) between observers on the presence of seven hazards, strong agreement (kappa between 0.8 and 0.9) on the presence of six hazards and very strong or perfect agreement (kappa> 0.9) concerning the remaining 22 hazards. Average Kappa index of agreement values for the standard observation items (mean 0.91) versus those observation items specific to urban / peri-urban areas (mean 0.90) were very similar. Kappa values were on average somewhat lower (0.89) for the 20 hazards relating to the water point head and its immediate surroundings (e.g. problems with cement at wellhead or pump), compared to the average (0.94) for the remaining observations of the wider environment (e.g. proximity to latrines or other hazards).

**Table 1:** Agreement between two observers concerning observations of contamination hazards at 62 water points in Greater Accra

Hazard	*A*	*B*	*C*	*D*	Overall agreement (%)	Kappa
Standard hazards						
Cement apron narrow/missing	43	18	1	0	98.39	0.962
Ponding on cement	37	23	0	2	96.77	0.932
Cracks/holes in cement	50	9	1	2	95.16	0.828
Loose pump	2	7	1	0	90.00	0.737
Ponding beyond cement floor	34	27	1	0	98.39	0.967
Drainage channel problems	52	6	1	3	93.55	0.714
Animal access	50	10	0	0	100.00	1.000
Fencing problems	59	1	0	1	98.36	0.659
Nearby latrines	20	26	0	1	97.87	0.957
Several nearby latrines	13	19	0	0	100.00	1.000
Latrine on high ground	21	25	0	0	100.00	1.000
Open water nearby	8	32	0	1	97.56	0.926
Uncapped wells nearby	7	29	0	0	100.00	1.000
Waste dump nearby	36	16	0	3	94.55	0.875
Unsanitary well cover	49	13	0	0	100.00	1.000
Well cover cracks/holes	41	12	0	2	96.36	0.900
Sloping well site	38	24	0	0	100.00	1.000
Well shaft sealing problems	47	7	0	1	98.18	0.923
Receptacle unsanitary	11	51	0	0	100.00	1.000
Urban/peri-urban hazards						
Sewage pipe nearby	29	31	1	0	98.36	0.967
Sewage pipe problems	7	54	1	0	98.39	0.924
Blocked storm drains	30	32	0	0	100.00	1.000
Faecal matter present	24	31	0	2	96.49	0.929
Diapers present	17	38	1	1	96.49	0.919
Polythene present	47	6	2	2	92.98	0.709
Kitchen waste present	31	20	4	2	89.47	0.782
Algae present	50	6	1	0	98.25	0.913
Dead rodents present	14	43	0	0	100.00	1.000
Paper waste present	44	11	2	0	96.49	0.895
Laundry washing nearby	56	5	0	1	98.39	0.900
Carwashing nearby	9	53	0	0	100.00	1.000
Farming nearby	12	49	1	0	98.39	0.950

Kappa index of agreement *p* value < 0.001 for all observed hazards

*A* hazard recorded by both observers, *B* hazard absent according to both observers, *C* hazard recorded by observer 1 but not observer 2, *D* hazard recorded by observer 2 but not observer 1

**Fig. 4:**
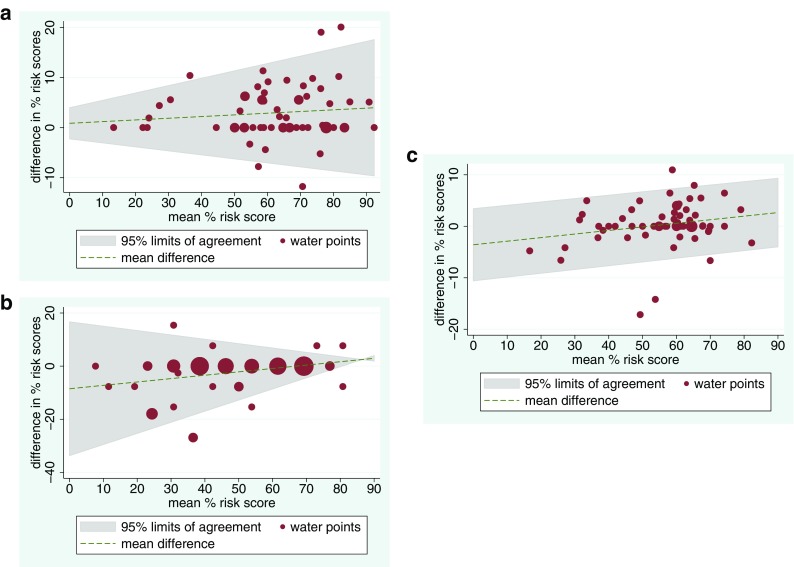
Bland and Altman plots of the difference in percent sanitary risk scores versus the mean percent risk score for both observers (grey area indicates 95% limits of agreement; *n* = 62 water points, with circle sizes proportional to number of water points) for: **a** standard WHO-derived observation items, **b** observation items specific to urban/peri-urban environments, and **c** both sets of observation items combined

Table 2 summarises the level of agreement in the percentage sanitary risk scores calculated from the first and second observers’ fieldwork. Overall, for the urban hazard scores, the standard protocol scores and these two scores combined, there was no evidence that one observer consistently observed more hazards than the other. The average difference in risk scores was 3% or less for all three sets of observation items. Lin’s concordance correlation coefficient, which measures both bias (consistent under- or over-scoring by one observer) and the correlation between the two sets of scores, was close to one, suggesting strong agreement. Pearson’s *r*, the correlation between the two observers’ hazard scores, was also very close to one. The Bradley-Blackwood *F* test suggested that the variance or mean difference in risk scores varied depending on the magnitude of the score for the urban observation items and the standard WHO items, but not the two combined. Evidence for this can be seen in Fig. 4, which shows Bland and Altman plots of the difference between observers’ scores versus the mean of their two scores for all water points. For standard WHO observation items, the limits of agreement, reflecting the difference in scores between observers, were wider for more hazardous water points with higher mean risk scores. For the urban observation items, the reverse was true, with wider limits of agreement for less hazardous, lower-scoring water points.

**Table 2:** Concordance correlation coefficients and related inter-observer agreement statistics for percentage sanitary risk scores recorded by two independent observers for 62 water points in Greater Accra

	Standard WHO observation items	Observation items for urban/peri-urban areas	All observation items combined
Lin’s concordance correlation coefficient (95% confidence intervals)	0.938 (0.901 to 0.962)	0.912 (0.862 to 0.945)	0.949 (0.917 to 0.968)
Pearson’s *r*	0.952 (*p* < 0.001)	0.927 (*p* < 0.001)	0.951 (*p* < 0.001)
Bias correction factor	0.985	0.984	0.997
Average difference in percent risk scores (95% limits of agreement)	2.95 (− 7.61 to 13.51)	− 2.40 (− 16.83 to 12.04)	0.28 (− 8.26 to 8.83)
Correlation between difference in risk scores and mean	0.107	0.311	0.214
Bradley-Blackwood *F* test	9.61 (*p* < 0.001)	6.798 (*p* = 0.002)	1.58 (*p* = 0.215)

Urban-specific risk scores were not significantly correlated with risk scores derived from the standard protocol for either observer (*r* = 0.11, *p* = 0.41 for observer 1; *r* = 0.16, *p* = 0.22 for observer 2).

### Changes in inter-observer agreement over time

**Fig. 5:**
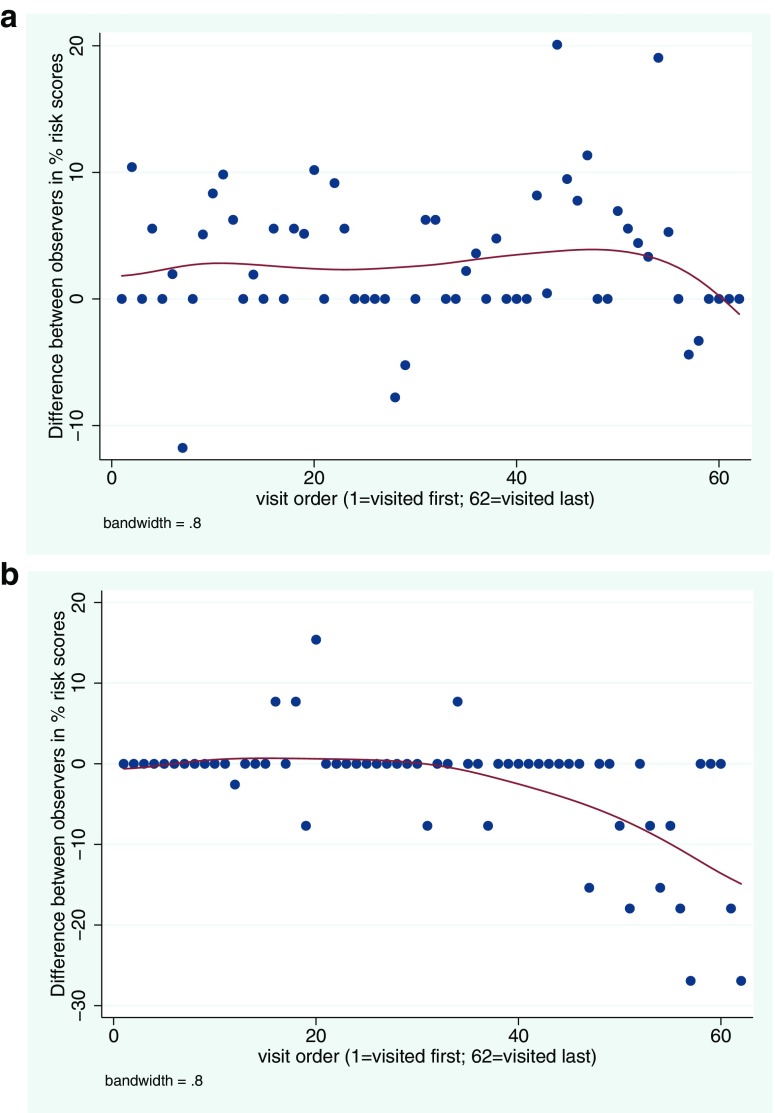
Difference in percentage sanitary risk scores recorded by two observers for 62 water points in the Greater Accra region, shown in relation to the sequence in which water points were visited for **a** standard WHO-derived observation items and **b** observation items specific to urban/peri-urban areas (the red line is derived via locally weighted scatterplot smoothing (LOWESS))

Figure 5 shows how the difference between the two observers’ risk scores varied in relation to the order in which water points were visited, with the urban/peri-urban-specific observation items shown separately from those items from the standard WHO protocol. Spearman’s rank correlation indicated no association between the order in which water points were visited and difference between observer risk scores for the standard WHO observation items (rho = − 0.05; *p* = 0.67), but a significant association for the urban/peri-urban observation items (rho = − 0.47; *p* < 0.001).

### Water point construction and sanitary risk

**Fig. 6:**
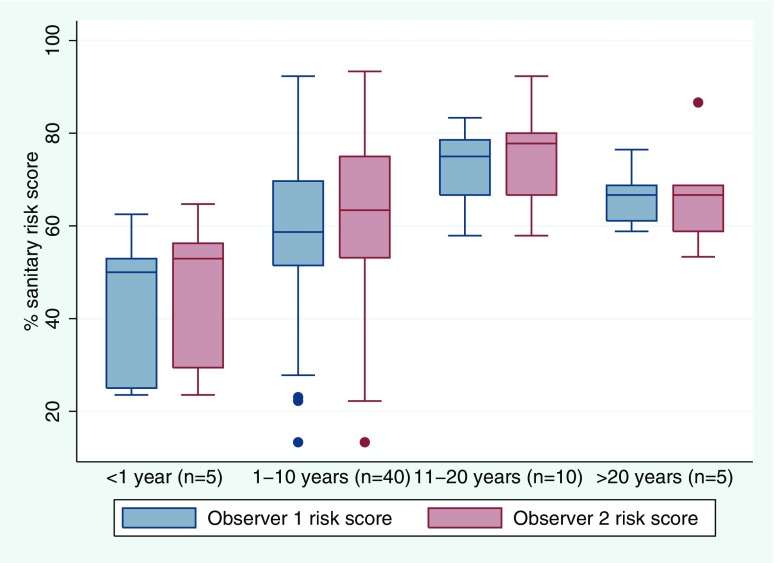
Relationship between sanitary risk scores (based on the standard WHO observation protocol) from two independent observers and years since installation of water point

For both observers, one-way ANOVA indicated a significant association between water point age and sanitary risk scores based on the standard WHO observation checklist (*F* = 4.47, *p* = 0.007 for observer 1; *F* = 3.70, *p* = 0.017 for observer 2), but not for the urban/peri-urban sanitary risk scores (*F* = 0.24, *p* = 0.865 for observer 1; *F* = 0.02, *p* = 0.996 for observer 2). This relationship is shown for the standard checklist in Fig. 6, which suggests lower risk scores for water points installed in the past year.

## Discussion

The concept of reliability is frequently used in public health and social sciences (Heale and Twycross 2015) to assess the extent to which quantitative measurements are replicable, but is seldom used in developing countries (Bolarinwa 2015). Much of the literature on sanitary risk inspection examines the relationship between risk scores or observed hazards and faecal indicator bacteria, e.g. Snoad et al. (2017), thereby focussing on the validity of observations rather than their reliability. Whilst there are published replicability assessments of other public health-related observation protocols of the living environment, such as the NIfETy method for assessing neighbourhood indicators of violence, alcohol and drugs (Furr-Holden et al. 2008), to our knowledge, our study is the first time such an assessment has been conducted for sanitary risk inspection protocols. Generally, levels of agreement between the hazards recorded by the two observers in our study were high (McHugh 2012), as shown by the Kappa index of agreement values in Table 2. One might expect there to be greater differences between the two sets of observations for hazards that are inherently difficult to inspect at first hand in densely packed urban settlements, such as the presence of a pit latrine within 30 m. However, there was no evidence of this in Table 1, with for example a loose-fitting pump being an observation item with a comparatively low kappa index of agreement value.

A lack of replicability of sanitary risk observations could have several consequences. For example, such lack of replicability could potentially weaken the strength of the observed association between contamination hazards and faecal indicator bacteria in groundwater, as undertaken in several studies (Howard et al. 2003; Godfrey et al. 2006; Misati et al. 2017). Similarly, it could lead to ambiguity over corrective action to reduce hazards (such as wellhead repairs), either for an individual water point or in prioritising water sources for remediation (Cronin et al. 2006) from amongst those found within a given area. Analogously to anthropometric standardisation in child nutrition surveys (de Onis 2006), such an assessment of inter-observer replicability could be incorporated into the training phase of larger-scale water point mapping surveys, to identify potentially ambiguous observation items, increase robustness of observations and so strengthen data collection efforts. We found some evidence that inter-observer agreement declined over time (Fig. 3b), suggesting that quality control measures for such surveys should also assess any evidence of observer fatigue. Given growing availability of camera phones in developing countries, there may be scope to photograph water sources, given that photography has been found to lessen observer fatigue when conducting other forms of environmental monitoring (van Dongen et al. 2017).

We found that risk scores for urban/peri-urban-specific hazards such as blocked storm drains or leaking sewage pipes were uncorrelated with risk scores derived using the standard WHO protocol (World Health Organization 1997). Inter-observer agreement was similar for the urban/peri-urban hazard observations and those based on the standard protocol (Table 2), with some urban hazards such as blocked storm drains and signs of ‘flying toilet’ defecation being observed around almost half of the selected water points. This suggests that such observations are replicable and capture hazards that are widespread in the case study urban and peri-urban areas, which would otherwise be missed by the standard observation protocol, were this generic set of observation items to be used without adaptation, as has happened in some studies (Mushi et al. 2012). This would suggest that nationwide sanitary risk inspection exercises, such as implemented in six countries through the Rapid Assessment of Drinking Water Quality surveys (World Health Organization and UNICEF 2012; Bain et al. 2012), should include separate observation checklists for urban and peri-urban versus rural areas. Given high density settlement patterns in urban and peri-urban areas, hazards around water points such as uncollected solid waste or leaking sewage pipes may be difficult for urban water point owners to remediate (Okotto-Okotto et al. 2015). Furthermore, some studies have suggested that other contamination risk factors such as local hydrogeology, wellhead construction (Ercumen et al. 2017) and propensity to flooding (Luby et al. 2008) are better predictors of faecal contamination of groundwater than such hazards in the environment around water points. If this is the case, then the omission of urban hazards from the standard sanitary risk observation protocol may not be problematic.

In our study, we found that sanitary risks at the water point head or well shaft were more frequently identified in older water sources (Fig. 3). This reflects a decline in water point functionality with age found in Ghana (Fisher et al. 2015) and also in three other sub-Saharan African countries (Foster 2013). This could reflect difficulties in maintaining water points, but potentially also improving standards of water point installation over time.

Our study is subject to several limitations. We examined reliability of sanitary risk inspection, not its validity (Heale and Twycross 2015), so our study provides no insights into the ability of the observation protocol to capture faecal contamination risk. Given the capacity-building nature of the project funding, both observers were studying for an educational qualification in public health. Thus, their educational backgrounds differed from the community-based water supply managers or field survey enumerators who might typically be expected to make such observations. Similarly, although we endeavoured to prevent collusion over observations via separate data entry and each observer inspecting water points whilst the other was engaged in another task, our study protocol did not include measures to prevent this from happening. This may result in over-estimation of inter-observer agreement. Conversely, the fact that one observer (WM) interviewed the water point owner or manager whilst the other did not may have resulted in systematic differences between the two sets of hazard observations. There was however no evidence of this in our limits of agreement analysis of hazard scores. All three limitations could readily be addressed via a subsequent study with assessment of replicability as its principal objective. Our findings are not applicable to rural areas, where lower settlement density will make observations of hazards surrounding water points easier. Because our study’s primary objective was to assess the relationship between sanitary risk scores and groundwater quality, we did not plan our desired sample size based on a test of inter-observer agreement. However, it would be straightforward to do so (Reichenheim 2001) in a future study where this was the primary objective. It would also be possible to expand the study design to include more than two observers and perform more sophisticated analysis of inter-observer agreement.

## Conclusion

Although many studies have used sanitary risk inspection to measure hazards that could lead to water source contamination, the reliability of sanitary risk inspection protocols has not previously been examined. Our study is the first to examine this issue, finding high agreement between hazards recorded by two different observers for groundwater sources in urban and peri-urban Accra. This suggests that sanitary risk inspection is sufficiently reliable to identify potential contamination hazards requiring remediation around water sources. Low or non-existent correlations between sanitary risk scores and microbial contamination have been found in many previous studies. This lack of correlation could be caused by low reliability in sanitary risk scores. Because our study found high reliability in risk scores, this points towards other explanations for the lack of an observed relationship between sanitary risk scores and microbial contamination, such as one-off microbial testing failing to capture transient contamination events. The methodology used in our study could be adopted in future large-scale water source surveys to quantify inter-observer variation in hazard recording. Sanitary risk scores based on an unmodified, standard protocol were uncorrelated with risk scores derived from urban contamination hazards. Thus, the unmodified, generic observation protocol did not capture contamination hazards commonly found in urban areas, such as discarded diapers or leaky sewerage pipes. This suggests that in future water source surveys, sanitary risk protocols should also be adapted to account for the specific hazards present in urban areas.
